# Editorial: Advances in pre- and postharvest applications to reduce qualitative and quantitative food loss and waste

**DOI:** 10.3389/fpls.2023.1149358

**Published:** 2023-02-10

**Authors:** Hossam S. El-Beltagi, Aditya Parmar, Tong Chen, Mohamed M. El-Mogy

**Affiliations:** ^1^ Agricultural Biotechnology Department, College of Agriculture and Food Sciences, King Faisal University, Al-Ahsa, Saudi Arabia; ^2^ Biochemistry Department, Faculty of Agriculture, Cairo University, Giza, Egypt; ^3^ Natural Resources Institute, University of Greenwich, Kent, United Kingdom; ^4^ Key Laboratory of Plant Resources, Institute of Botany, Chinese Academy of Sciences, Beijing, China; ^5^ Vegetable Crops Department, Faculty of Agriculture, Cairo University, Giza, Egypt

**Keywords:** quality, post-harvest, food decay, horticulture crops, shelf-life, storage

Global demand for high-quality fresh fruits and vegetables is increasing rapidly, thanks to a rising middle class, urbanisation, an increase in disposable income, and changing consumer habits. The global market for fresh fruits and vegetables is estimated to be 144 billion USD, which is anticipated to increase to more than 200 billion USD by 2027 (Stanaway et al., 2022). Fruits and vegetables are essential to a healthy and well-balanced diet. They are abundant in essential vitamins, antioxidants, minerals, and dietary fibres, which can help with a range of ailments and deficiencies (Chen et al., 2022).

Fresh fruits and vegetables are highly perishable and are spoiled due to many factors that occur post-harvest. The quality of fruits and vegetables and their storage ability after harvest are affected by many pre-harvest factors such as fertilisation, irrigation, soil type, planting distances, and many other factors. On a global scale, a significant amount (25-50%) of fruits and vegetables are lost from farm to fork, which is referred to as post-harvest losses or Food Loss and Waste (FLW). This loss represents about a third of the amount of food produced in the world (Bancal and Ray, 2022). Moreover, the primary challenge for agricultural research and policy is to feed more than 9.1 billion people with safe food by 2050. As a result, food production is expected to increase by 60% by 2050 to meet the world’s food supply needs (Parfitt et al., 2010). Based on the global challenge of FLW, we organised this Research Topic “Advances in Pre- and Post-harvest Applications to Reduce Qualitative and Quantitative Food Loss and Waste”. The topic will enhance knowledge and awareness about the pre-and post-harvest treatments that could help reduce the global FLW of fresh fruits and vegetables.

A total of six post-harvest treatments (articles) have been tested to evaluate their effects on the storage ability of different crops. Hassan et al. study the effect of different modified atmosphere packages (non-perforated polyethylene, polypropylene packets, brown paper bag, and without packaging) on the quality and nutritional values of the pointed gourd (*Trichosanthes dioica* Roxb.) under either low- temperature (4°C) or room- temperature (30°C) conditions. The results reveal significant differences between the treatment variables in all dependent parameters studied for low- and room-temperature storage conditions. Perforated and non-perforated polyethylene and polypropylene packaging retained a significant amount of b-carotene, Vit. C., and greenish colour (lower L*and high h*) in the pointed gourd after storage at low and room temperatures. Furthermore, the principal component analysis (PCA) revealed some quality attributes such as L*, C*, h*, shelf-life, and Vit. C. were significantly influenced in non-perforated polyethylene packaging under both conditions. In conclusion, a non-perforated polythene package would be the best condition for retaining the market quality and acceptance appearance of the pointed gourd for up to 23 and 10 days in the refrigerator and ambient storage conditions, respectively.

Liu et al. evaluate the post-harvest performance of papaya fruits when immersed in different ethanol solutions (0, 12.5, 25, 50, and 100 ml/L) for 2 hours and kept at 25°C for 14 days. The results suggest that ethanol soaking treatment maintains papaya fruits’ quality by delaying colour change, reducing decay incidence and water loss rate, and slowing firmness decline. Additionally, ethanol treatment enhanced the antioxidant system and postponed the maturity of papaya after harvest. This was achieved by decreasing the formation of malondialdehyde, ethylene, and superoxide anions, boosting the activities of superoxide dismutase, catalase, and ascorbate peroxidase, and inhibiting those of peroxidase, phenylalanine ammonia-lyase, and polyphenol oxidase. The authors suggest that treatment with 25 mL/L ethanol is the best treatment for papaya since it can potentially delay ripening and retain the fruits’ storage quality.

Wu et al. study the effect of ozone fumigation on the quality and microbial decay of blueberry fruits in China. The results demonstrate that ozone fumigation inhibited *Penicillium* sp. grown on blueberry fruits stored at 4°C for 80 days. Additionally, ozone fumigation treatment could maintain fruit firmness, and delay the loss of total phenolics, soluble solids, and anthocyanins. Also, lower polyphenol oxidase and greater peroxidase and catalase activity levels could be achieved by ozone fumigation.

Nxumalo et al. evaluate the effectiveness of green synthesised zinc oxide nanoparticles (ZnO-NPs) carried by Arabic gum in maintaining the post-harvest quality of mandarin fruits. The fruits were dipped in edible coating compounds for 3 min. and stored at 5°C and 95% relative humidity for 40 days. Their results show that ZnO-NPs carried by Arabic gum as an edible coating were the most effective treatment for reducing physiological disorders and maintaining the quality of mandarin fruits (cv. Kinnow). Also, ZnO-NPs at a rate of 0.5% + Arabic gum significantly reduced weight loss, chilling incidence, and electrolyte leakage compared to the control. However, the rate of 1% ZnO-NPs reduced the incidence of rind pitting. Finally, all treatments preserved the phytochemical and antioxidant content of fruits and enhanced the activity of antioxidant enzymes compared to control samples under cold storage conditions.

It is still unknown how chitosan would affect the reactive oxygen species metabolism of mechanically damaged apples during storage. Thus, Ackah et al. study the effects of post-harvest chitosan treatment on reactive oxygen species production and enzymatic and non-enzymatic antioxidant systems of the wounded apple fruits under storage conditions. Apple fruits were artificially wounded, then treated with 2.5% chitosan and kept at 21–25°C and 81–85% RH for seven days. Their results demonstrate that chitosan treatment increased the production of superoxide anions and hydrogen peroxide in fruit wounds and enhanced the activities of NADPH oxidase and superoxide dismutase. In contrast to the damaged control fruits, the chitosan-treated fruits showed a considerable reduction in malondialdehyde, lipoxygenase, and membrane permeability.

Additionally, chitosan treatment enhanced peroxidase and catalase activities in the wounded fruit. Also, their results show that wounding stimulated the production of reactive oxygen species. In conclusion, chitosan treatment after harvest promises to be a valuable preservative to maintain fruit quality.

Finally, Rutta et al. focus on assessing a low-cost technology (solar-powered cold storage) for extending the shelf-life of tomato fruits in Tanzania for small-scale farmers. The authors mention that approximately 50% of total tomato production is lost due to poor post-harvest practices. One of the main reasons for the enormous post-harvest tomato losses is the absence of cold storage facilities, which has a detrimental effect on farmers’ livelihoods and the sector’s economic contribution. Thus, their study looks into Tanzania’s challenges in adopting and deploying solar-powered cold storage technologies. The results show that the deployment of solar-powered cold storage technologies is limited by factors such as high investment costs, partial knowledge, low-paying capacity among farmers, and non-refrigerated foods that are preferred by consumers.

Previous research shows the importance of post-harvest treatments in reducing the percentage of loss and damage, increasing the number of products, and reducing FLW globally.

## Author contributions

ME-M wrote the article. HE-B, AP, and TC revised the manuscript. All authors contributed to and approved the article.
